# Pump Up the O_2_: Is Supersaturated Oxygen Therapy the Holy Grail to Reduce Infarct Size in Anterior STEMI?

**DOI:** 10.1016/j.jscai.2026.105440

**Published:** 2026-05-07

**Authors:** Benjamin Z. Galper

**Affiliations:** Mid-Atlantic Permanente Medical Group, McLean, Virginia

**Keywords:** cost-effectiveness, ST-elevation myocardial infarction, supersaturated oxygen

Despite increasing success in the race to rapidly open occluded coronary arteries in patients with anterior ST-elevation myocardial infarction (STEMI), morbidity and mortality remains high, with an incidence of heart failure of at least 10% at 1 year post-myocardial infarction (MI) and a direct correlation between infarct size and risk of developing heart failure post-MI.^1^ Reducing infarct size in anterior MI has thus become the holy grail in interventional cardiology, yet multiple approaches—stem cell infusions, ischemic preconditioning, and use of mechanical support devices during STEMI interventions—have not demonstrated significant benefit.^1^, ^2^, ^3^ Supersaturated oxygen delivery (SSO_2_) at the time of STEMI has the potential to significantly reduce infarct size, but despite evidence that it may reduce infarct size by 24%, the use of SSO_2_ has been relatively limited.^4^^,^^5^ In this issue of *JSCAI*, Vilain et al^6^ demonstrate the first cost-effectiveness analysis of SSO_2_ to reduce infarct size in anterior STEMI.

Although SSO_2_ delivery at the time of STEMI has been shown to reduce infarct size, the use of SSO_2_ has not penetrated regular clinical practice, and despite US Food and Drug Administration approval, SSO_2_ administration has not been included in American College of Cardiology/American Heart Association guidelines for the treatment of acute coronary syndromes. Vilain et al constructed a rigorous cost-effectiveness analysis of SSO_2_ in anterior STEMI and demonstrated that SSO_2_ is cost effective compared with standard STEMI care, with an incremental cost-effectiveness ratio of only $38,415 per quality-adjusted life year gained, well within the societal willingness-to-pay threshold. The authors included a number of sensitivity analyses around potential uncertainty in model input parameters and concluded that the probably of SSO_2_ providing good economic value is 73%.

Although the model results are compelling, a cost-effectiveness model is only as good as its data inputs. On deeper investigation, the randomized data on SSO_2_ benefit in anterior STEMI is only based on 2 relatively small trials. The AMIHOT I trial randomized 269 patients with either anterior or large inferior STEMI to SSO_2_ vs standard of care, with the primary end point being infarct size. The overall trial results were negative, but a subgroup analysis of 105 patients with an anterior MI treated with SSO_2_ demonstrated a statistically significant decrease in infarct size of 14%.^7^

The AMIHOT II trial randomized 222 patients with successful percutaneous coronary intervention for anterior STEMI and demonstrated only a 6.5% difference in infarct size, which was statistically significant. When these patients were pooled with the anterior MI patients from AMIHOT I, the analysis demonstrated a 24% relative reduction in infarct size with SSO_2_ compared with standard therapy.^4^ Additionally, SSO_2_ seemed to substantially benefit patients with larger infarcts and lower baseline ejection fraction, with only a 4% decrease in infarct size for small anterior MIs and a 10% infarct size reduction in patients with baseline ejection fraction <40%. Given that the inputs for the cost-effectiveness model were essentially based on 2 relatively underpowered trials and a total of 491 patients, one must take pause before claiming that SSO_2_ is definitively cost effective.

The beauty of a well-constructed cost-effectiveness analysis is not merely in running the base case simulation but in anticipating the plausible variations in key data points that lead to uncertainty in the model outputs. The authors performed a number of sensitivity analyses including multiple 1-way sensitivity analyses, in which the most important model parameters were modeled around plausible ranges of their true value, and each parameter’s impact on the incremental cost-effectiveness ratio of SSO_2_ was demonstrated in a tornado diagram. The tornado diagram demonstrated that the model inputs most likely to lead to SSO_2_ not being cost effective are a lower than expected infarct size without SSO_2_, an older patient age at the time of MI, a lower hazard ratio for heart failure incidence per each 1% increase in infarct size, and a lower relative reduction of infarct size with SSO_2_ therapy. The authors accounted for this uncertainty through probabilistic sensitivity analyses in which all model parameters were varied around plausible input ranges for over 2000 model iterations and determined in 73% of simulations, SSO_2_ therapy for all-comers is likely to be cost effective. Regardless of potential uncertainty in the model inputs, it seems quite certain that in younger patients with large anterior MIs and with larger infarct sizes that SSO_2_ is very likely to be cost effective. In fact, the more contemporary IC-HOT study demonstrated that SSO_2_ in young patients (mean age 60 years) with large anterior MIs led to improved 1-year clinical outcomes compared with a propensity-matched cohort.^5^

Although Vilain et al demonstrated that SSO_2_ is likely cost effective for anterior STEMI patients, the relative lack of penetration of SSO_2_ into standard clinical practice at the time of STEMI cannot be minimized. Treatment of a STEMI is a fast-paced procedure performed as an emergency, frequently on off hours when every minute counts. The STEMI team is likely an on-call team with little support in the middle of the night. SSO_2_ requires additional equipment, including a larger 7F femoral sheath, and the SSO_2_ therapy itself takes 60 minutes to complete. Given that radial access has become the preferred approach for STEMI^8^ and that significant extra procedure time for an on-call team is typically discouraged, the uptake of SSO_2_ faces multiple potential logistical barriers. Even intravascular ultrasound, which takes a mere few extra minutes per case and has been demonstrated to lead to improved procedural outcomes, has faced substantial barriers to widespread intervention, with only 15% of US percutaneous coronary interventions using it.^9^ Even if cost effective, SSO_2_ faces an uphill climb before widespread implementation.

Decreasing infarct size has been the elusive holy grail for STEMI treatment. Although multiple approaches to reduce infarct size in the periprocedural and intraprocedural STEMI setting have failed to show promise, SSO_2_ demonstrates that it is likely to reduce infarct size and be particularly cost effective in larger anterior MIs. Especially if SSO_2_ can be delivered via radial access, clinical leaders across the United States should begin to consider mechanisms to pump up the oxygen in the catheterization laboratory and introduce SSO_2_ therapy into the STEMI algorithm.
